# High‐Sensitivity RFID Sensor for Structural Health Monitoring

**DOI:** 10.1002/advs.202301807

**Published:** 2023-07-05

**Authors:** Hussein Nesser, Hassan A. Mahmoud, Gilles Lubineau

**Affiliations:** ^1^ Mechanical Engineering Program Physical Sciences and Engineering Division King Abdullah University of Science and Technology (KAUST), Physical Science and Engineering Division Thuwal 23955‐6900 Saudi Arabia; ^2^ Mechanics of Composites for Energy and Mobility Laboratory King Abdullah University of Science and Technology Thuwal 23955 Saudi Arabia

**Keywords:** chipless sensors, radio frequency identification devices, strain sensors, structural health monitoring, wireless strain monitoring

## Abstract

Structural health monitoring (SHM) is crucial for ensuring operational safety in applications like pipelines, tanks, aircraft, ships, and vehicles. Traditional embedded sensors have limitations due to expense and potential structural damage. A novel technology using radio frequency identification devices (RFID) offers wireless transmission of highly sensitive strain measurement data. The system features a thin, flexible sensor based on an inductance‐capacitance (LC) circuit with a parallel‐plate capacitance sensing unit. By incorporating tailored cracks in the capacitor electrodes, the sensor’s capacitor electrodes become highly piezoresistive, modifying electromagnetic wave penetration. This unconventional change in capacitance shifts the resonance frequency, resulting in a wireless strain sensor with a gauge factor of 50 for strains under 1%. The frequency shift is passively detected through an external readout system using simple frequency sweeping. This wire‐free, power‐free design allows easy integration into composites without compromising structural integrity. Experimental results demonstrate the cracked wireless strain sensor's ability to detect small strains within composites. This technology offers a cost‐effective, non‐destructive solution for accurate structural health monitoring.

## Graphical abstract

This work proposes a wireless strain sensor for structural health monitoring (SHM) in composites, using radio frequency identification (RFID) and a cracked design. It achieves high sensitivity, eliminates wires and power sources, and integrates seamlessly. Experimental results confirm its effectiveness in detecting small strains, ensuring safety.

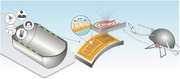

## 1 Introduction

Monitoring composite structures is essential for detecting critical events and severe degradation during operations. Several nondestructive testing (NDT) methods are available for detecting damage and defects inside composite materials, such as the ultrasonic testing method,^[^ ^1^ ^]^ eddy current testing,^[^ ^2^ ^]^ shearography^[^ ^3^ ^]^ and radiographic testing,^[^ ^4^ ^]^ infrared thermography,^[^ ^5^ ^]^ and optical testing.^[^ ^6^ ^]^ All these existing NDT methods have limitations in some of the basic requirements for composite monitoring systems, such as prohibitive cost, limited lifetime, difficulty of integration, challenging large‐scale coverage, incompatibility with harsh environments, reliability, flexibility, and multifunctionality.^[^ ^7^, ^8^ ^]^ One of the proposed solutions is realizing a “smart structure” by equipping the structural materials with sensing capabilities.^[^ ^9^, ^10^, ^11^, ^12^, ^13^ ^]^ Currently, the deployment of sensors is often limited by wired connections that result in installation problems and costs. Accordingly, embedded sensors that can communicate wirelessly for both data and energy transmission are an important research direction.^[^ ^14^ ^]^

Because of their unique communication feature, Radio‐frequency identification (RFID) sensors have been used in various applications, including healthcare, food quality, agriculture, and space.^[^ ^15^, ^16^, ^17^, ^18^ ^]^ Among this wide group of RFID sensors, passive RFID tags do not contain an internal power supply; therefore, they harvest all the energy required from the RFID reader. However, such passive RFID sensors are chipless, which makes them very compact and possibly nonintrusive; thus, they represent a promising technology for creating smart composites. Moreover, the lifetime of such a system will be longer than those of battery‐dependent active systems. In addition, RFID tags can be fabricated at a very low cost, which allows high‐resolution monitoring of large systems, such as pipes and tanks.

Considerable research, not necessarily employing chipless systems, has been conducted to develop RFID sensing systems for SHM.^[^ ^19^, ^20^, ^21^ ^]^ For example, a patch antenna is widely used to detect cracks and strain in structures. Marindra et al. proposed a chipless RFID sensor tag integrating four tip‐loaded dipole resonators as a four‐bit ID encoder and a circular microstrip patch antenna resonator as a crack sensor that could detect the orientation of the cracks in metallic structures.^[^ ^22^ ^]^ Alternatively, a carbon fiber microstrip patch antenna was presented by Preddy et al.; this sensor was constructed entirely from fiber‐reinforced plastic (FRP)‐based materials and fabricated directly into carbon FRP (CFRP) composites as part of the composite‐manufacturing process.^[^ ^23^ ^]^ The Institute for Microsensors, Actuators, and Systems at the University of Bremen developed an embedded wireless pressure and temperature sensor for monitoring resin flow during the fabrication processes of glass and carbon fiber composites.^[^ ^24^, ^25^, ^26^ ^]^ As a direct application in SHM, Zarifi et al. developed a chipless RFID‐based sensor for pipeline integrity monitoring in real‐time.^[^ ^27^ ^]^

**Figure 1:**
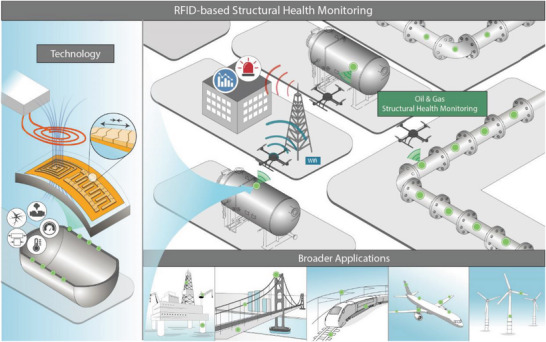
Supersensitive passive RFID strain sensor: applications and technology.

Eliminating all rigid components (wires, battery, and chip) from the sensor packaging improves the flexibility and integrability of composites, which positively influences measurement reliability and performance compared to rigid or partially rigid sensors, which may affect material properties. Chipless RFID sensor‐based inductor–capacitor (LC) oscillators exhibit tremendous potential for extensive use as a new SHM component for composite structures. However, the strain to failure in composites is usually very small (the rupture strain of main composite materials, such as CFRP, glass FRP (GFRP), and aramid FRP ranges between 1,5% and 2,5%), requiring a sensitive sensor capable of detecting small strains (<1%). However, all the available chipless RFID sensors exhibit low sensitivity, as summarized in Table S1 (Supporting Information), which presents some existing works on chipless sensors in various applications. The sensitivity of chipless RFID sensors should be improved before they become a viable type of sensor in composite SHM (CSHM). Here, we present a wireless and batteryless sensor for monitoring strain in composite structures (**Figure** **1**). Our sensor introduces a significant advancement in the field of strain sensing, particularly in its high sensitivity and wireless detection capability for very small strains. This breakthrough sets it apart as the first sensor capable of wirelessly monitoring composites even before they reach the cracking state. To better illustrate this, we have included a detailed comparison in Table S1 (Supporting Information), which showcases the performance and limitations of conventional chipless wireless strain sensors in terms of sensitivity and resolution. By highlighting the unique aspects of our approach and emphasizing the limitations of existing sensors, we aim to underscore the novelty and significance of our work. Moreover, our sensor holds tremendous potential in enhancing the safety and efficiency of composite materials across various applications. With its wireless detection capability for small strains, it provides early warnings for potential damage, enabling timely maintenance and repair, and reducing the risk of catastrophic failure. The chipless RFID sensor is a thin and flexible device that can be easily embedded between the interlayers of a structure. Wireless communication is realized using the radio frequency coupling method, where very simple and low‐cost equipment and interrogation system are required to collect sensor data. Our RFID sensor comprises a variable capacitance, which is affected by strain and connected to a coil to form an LC oscillator with a unique response frequency that changes with a change in the capacitance value. Its uniqueness is in the advantage of the electromagnetic signal damping along the capacitor through highly piezoresistive electrodes. The result is an ultrasensitive wireless strain sensor suitable for CSHM applications. The operation of the sensor is demonstrated through a lab test by bending an embedded sensor in a piece of GFRP sample, showing accurate strain monitoring in real time. Furthermore, the device made entirely of flexible materials with controllable dimensions can be deployed in a wireless sensor network (WSN) system with a finer spatial granularity for accurate monitoring and big data applications where wired sensors would have severe limitations (Figure 1).

## 2 Results and Discussion

### 2.1 Design of the RFID Strain Sensor with Piezoresistive Cracked Electrodes

**Figure 2:**
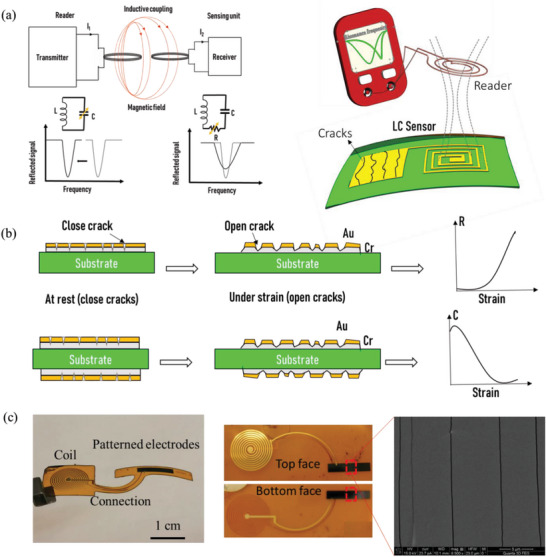
Cracked RFID sensor mechanism and design: a) Wireless communication between the transmitter antenna and the receiver sensor via inductive coupling. b) Existence of cracks in the electrodes, making them highly piezoresistive and resulting in a remarkable change in capacitance by changing the penetration of the interrogation signal. c) Photographic image showing the designed LC sensor, including the top (coil + top electrodes) and bottom (connection + bottom electrode) faces of the substrate. The scanning electron microscopy (SEM) image reveals the cracks existing in the electrodes.

An RFID system usually comprises a receiving coil and a transmitting coil (**Figure** **2**a). These two coils interact with each other via electromagnetic coupling. The alternative current flowing through the transmitter coil generates a variable magnetic flux, which creates a proportional electromotive force according to Faraday's Law (Figure 2a). In general, the chipless tag is composed of an inductor and a capacitor to form an inductor–capacitor (LC) oscillator circuit.^[^ ^28^ ^]^ The variations in the sensing unit properties induce a change in the reflected signal. We use capacitive sensing, where the change in capacitance is used to measure the external stimuli. However, the two main mechanisms for detecting a variation in the sensor response are the quality factor change (Δ*Q*) or the frequency shift (Δ*f*) in the reflection coefficient (Figure 2a). The shift in the resonance frequency of the oscillating circuit is due to the change in the capacitance of the sensor, and by knowing the capacitance variation of the sensor through the response frequency shifting, we can deduce the resistance variation through the quality factor. The quality factor can then be used to collect information from the sensor. Meanwhile, few studies have been devoted to monitoring structures with chipless LC sensors because of their limited ability to measure small stimuli, which is an important requirement in SHM. In particular, when measuring the shift in resonance frequency, the sensitivity of such sensors is guided only by the geometrical change in the capacitance under stretching, which usually leads to a gauge factor (GF) close to 1.^[^ ^29^ ^]^ Achieving high sensitivity has appeared very challenging and witnessed limited success thus far. We have been developing a new generation of capacitive/resistive sensors with a novel technology to monitor local deformation with high sensitivity.^[^ ^29^, ^30^, ^31^, ^32^ ^]^ First, we developed a novel crack‐based resistive strain sensor with extremely high sensitivity.^[^ ^31^ ^]^ The active area of this sensor comprises fragmented conductive films, which enable extremely high piezoresistivity compared to other families of resistive strain gauges (GF = ≈10^4^) (Figure 2b). In this work, we build electrodes that are also conductive plates or parallel‐plate capacitors, so that they feature a large change in resistance under stretching. This is a key enabling component of our high‐sensitivity capacitive technology. To drastically improve the GF of capacitive sensors, we integrated the developed high‐piezoresistivity electrodes in a classical parallel‐plate capacitor to form a transmission line model (Figure 2b), which resulted in different penetration levels of the interrogation signal over the sensor length.^[^ ^30^ ^]^ This design results in a strain‐ and frequency‐dependent capacitance with ultrahigh sensitivity that unlocks the potential of capacitive sensing. These crack‐based strain sensors showed extremely high sensitivity with GF = 37, demonstrated by using a cracked carbon nanotube paper as the electrode and polydimethylsiloxane as dielectric materials.^[^ ^30^ ^]^ With the same concept and to fit the framework of composite structure monitoring, we transferred this technology to a durable, flexible, and thin sensor through microfabrication technology using polyimide substrate and active metallic layers (Figure 2c). We combined the cracked capacitive sensor with an inductive coil to form a new type of wireless sensing method using the RFID technique. Figure S1 (Supporting Information) depicts the design of our metallic–polyimide LC sensors, in which we separated the coil from the sensing unit (capacitor) to avoid crack creation in the coil. The capacitance and coil geometries were selected through an analytical study to optimize the sensor performance (resonance frequency, electromagnetic response, and mutual coupling) and by considering the integration conditions. A 50 µm thick polyimide foil was used as the substrate and a dielectric material was used to separate the top and bottom electrodes to form a parallel‐plate capacitor (other types of materials with different thicknesses can be used). Two opposite rectangular electrodes with length and width of 10 and 3 mm, respectively, which formed the capacitive part, contained nanocracks to form a very sensitive sensing unit capable of measuring very small strains (Figure 2c and Figure S1, Supporting Information). The geometric parameters of the electrodes and the dielectric property of the flexible substrate define the initial capacitance *C*_0_, as given by Equation S1 (Supporting Information). Moreover, the coil loss factor and inductive coupling define the quality of the transmitted and reflected signal from and to the sensor coil. Hence, we selected a planar circular coil with a large cross‐section to optimize the sensor coil quality factor and the electromagnetic coupling according to Equations S1–S7 (Supporting Information). The simulated dimensions of the sensor component are presented in Table S2 (Supporting Information), affording a capacitance of 45 pf, inductance of 210 nH, and a coil resistance of 7,5 Ω. Equations S3 and S4 (Supporting Information) lead to an initial resonance frequency of ≈50 MHz and a coil loss factor of 8.8, which is enough for harvesting a sufficient amount of signal, circulating it in the circuit, and reflecting it back to the reader coil without losing all the signal inside the circuit. Table S3 (Supporting Information) summarizes the electrical characteristic of the designed sensor, which fits very well with the experimental measurement of the cracked sensors. The initial resonance frequency, which is dependent on the capacitance and inductance of the LC circuit, was chosen to be shiftable by the piezoresistivity of the electrodes.

### 2.2 Effect of Nanocracks in the Metallic Electrodes of the Capacitance

**Figure 3:**
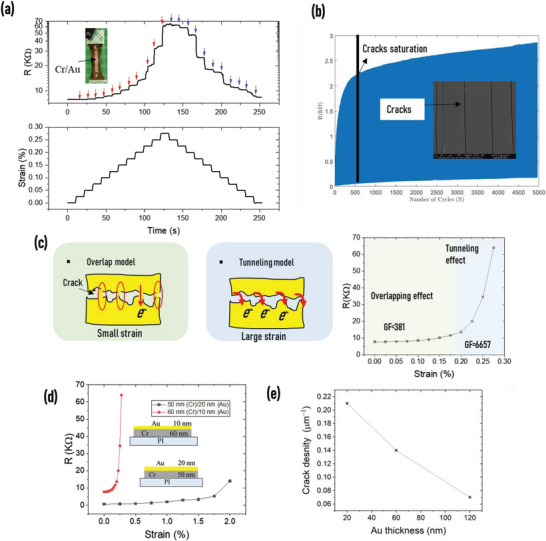
Electrical behavior of a cracked Cr/Au nanofilm on a PI substrate: a) Resistance variation of the cracked Cr/Au film under strain steps (red arrow for loading and blue arrow for relaxing). b) Crack creation by cycling, where the resistance represents the crack density, which increases under strain cycling before saturation; the SEM image clearly shows the cracks on the Cr/Au film. c) Overlapping and tunneling mechanisms of the electronic conduction between two conductive fragments, and the resistance variation under strain. d) Influence of the thickness of the Cr/Au layer on the resistance behavior. e) Crack density calculated from SEM images of three samples with different thicknesses of Au.

Figure 2c shows the crack‐based sensor and the cracks inside the electrodes. The cracked electrodes were fabricated with two superposed metallic layers: one to trigger the cracks and the other to ensure good electronic conduction. Cr, films of which are widely used as interlayers to promote the adhesion of conductive metals to substrates, is a rigid metal that promotes the formation of a network of cracks. However, a Cr interlayer usually becomes fragmented at low strains compared to ductile metal films. The Cr layer is covered by a gold layer (Au), which has good conducting properties, to ensure low resistance when the electrodes are not stretched. Cracking in the conductive layer creates a piezoresistive effect in the materials, resulting in resistance variation under strain while maintaining enough conductivity to still allow reduced (but nonzero) electronic conduction.^[^ ^33^ ^]^ **Figure** **3**a shows the resistance variation as a function of small strain steps of 0.05% for a cracked Cr/Au nanofilm. Two different mechanisms could be ascribed to the strain‐dependent resistive effect. In the first stage, an overlapping regime dominates, which is replaced by a tunneling regime in the second stage. Before the opening of the crack, electrical conduction occurs throughout the gold layer. Applying strain to the film opens the crack partially; thus, electrons can still flow through the overlapping regions. Referring to Figure 3c, the overlapping phenomenon dominates for a strain less than 1.8% with a GF of 381. When the overlapping regime ends, over a small distance (<70 nm) between the neighboring crack kips, electrons have the possibility of jumping through the opposite edges of a channel crack. Thus, the electrical conduction is ensured during that stage by the tunneling effect. The tunneling mechanism affects the electrical response over the strain range of 1.8–2.8%, affording a high GF of 6657. When stretching becomes high enough, the adjacent crack lips become totally separated, thus creating an open circuit.

The crack density, defined as the number of cracks per micrometer of length, determines the piezoresistivity level of the cracked electrodes. The more the number of cracks, the greater the resistance change. The crack density in the electrodes of the LC sensor is essential for realizing the excellent functionality of the transmission line model. In the transmission line mechanism, the interrogation frequency and electrode resistance have a similar effect on the signal dissipation in the capacitance.^[^ ^30^ ^]^ However, as an LC oscillating configuration is considered, the frequency cannot really be used to leverage this effect as we have to work in the vicinity of the resonance frequency of the circuit. This imposes a condition of designing with a specific range of electrode resistance. However, it is essential to control the resistance variation of the electrodes to activate the transmission line in the capacitor at the resonance frequency of the LC oscillator, as well as optimize the sensor sensitivity. As the signal attenuation speed is directly related to the electrode piezoresistivity, a strain range should be targeted to achieve efficient attenuation, which is realized by controlling the number of cracks in the electrodes. The fragmentation in the Cr/Au film is realized by strain cycling of the polyimide substrate. As shown in Figure 3b, the crack density—represented electrically by the resistance of the Cr/Au film—increases with the cycle number, saturating after a certain number of cycles. Static strain is sufficient for creating fractures in the chrome layers; in such a case, the crack density only depends on the thickness of the chrome layer. When the Au layer is added on top, propagation of the cracks needs cycling, where the saturation is related to the thickness of the Au layer. Design is important, as a slight change in the thickness ratio of the Cr/Au film induces a different resistance behavior of the Cr/Au film, as shown in Figure 3d. The 60 nm/10 nm film exhibited a high initial resistance *R*_0_ and GF of 7,6 KΩ and 2620, respectively. On the contrary, the 50 nm/20 nm film showed *R*_0_ and GF of 670 Ω and 390, respectively, implying that the resistance behavior of the electrodes under strain can be controlled by controlling the thickness ratio of the Cr/Au film. It should be noted that our current sensor configuration does not experience any adhesion problems since the gold film maintained strong adhesion even after undergoing multiple strain cycles. Furthermore, if necessary, additional techniques, such as interlayer deposition or surface roughness modification can be employed to improve adhesion between the metals and the substrate.

To study the effect of gold thickness on the crack density, we fixed the Cr thickness at 60 nm while varying the gold thickness. We observed the number of cracks and change in resistance after cycling. Results confirmed that the propagation of the cracks within the gold layer was related to its thickness, where increasing the gold thickness prevented the cracks created in the Cr layer from propagating. Then, the final state of the thick Cr/Au film was an electrode with a limited number of cracks (Figure S3, Supporting Information). Figure S4 (Supporting Information) shows SEM images of three samples with three gold thicknesses under 3000 times cycling at 5% strain; the number of cracks present in a zone of 100 µm is 21, 14, and 7 for 20, 60, and 120 nm, respectively, revealing a linear relationship between the crack density and the thickness (Figure 3e). For high crack density (high electrode piezoresistivity), the signal in the capacitance disappears very quickly at the resonance frequency, giving the sensor insufficient margin to shift its resonance frequency because it reaches the maximum level even before it is subjected to strain. Therefore, selecting the optimal metal thickness is necessary to obtain an effective sensor. However, low crack density in the electrodes prevents the electromagnetic signal attenuation at the resonance frequency, as the sensor needs a very high strain to activate the transmission line; therefore, the impact of the capacitance variation on the resonance frequency is very limited. On the basis of this understanding, we chose a Cr thickness of 60 nm and Au thickness of 100 nm to adapt the transmission line phenomena to the resonance frequency, where it recorded an initial resistance *R*_0_ = 396 Ω and an average variation of 220 Ω at 1% strain as shown in Figure S7 (Supporting Information).

### 2.3 Strain‐Sensing Mechanism: Electromagnetic Wave Penetration

**Figure 4:**
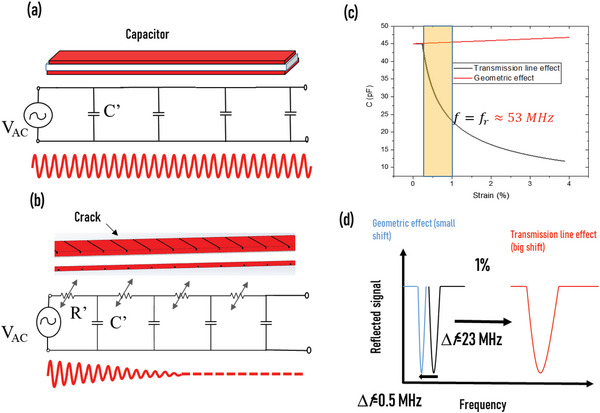
a) Equivalent circuit for a conventional capacitor in which the EM signal propagates in the entire structure because of the low‐resistance electrodes. b) The equivalent circuit for the cracked capacitance includes a variable resistance that causes EM signal damping in the structure body. c) Analytical study of the cracked and conventional capacitance, where the graph presents the variation of the capacitance under strain. d) Shifting of the resonance frequency of the LC circuit with classical and transmission line capacitive sensors.

We used the electromagnetic signal damping inside a parallel‐plate capacitance, representing the sensing component on the LC chipless sensors, to create an ultrasensitive strain sensor capable of detecting defects that occur underneath the surface of composite structures. Our concept is to take advantage of the piezoresistivity of the electrodes in the parallel‐plate capacitance to create a transmission line with variable resistance. The existence of resistance in the transmission line model transforms the pure capacitance into distributed R–C chains, particularly at high interrogation frequencies. **Figure** **4**a,b shows the effect of the electrode resistance on the electromagnetic wave propagation in the piezoresistive capacitor. For negligible electrode resistance, the sensing element behaves like a conventional capacitor, where the system is modeled as smaller capacitors distributed in parallel over an infinite number of segments (∆*l*); in this case, the EM signal propagates over the entire capacitance length without any damping (Figure 4a) even for high frequencies. The electrical model of the sensing unit is defined as a network of resistance and capacitance over the sensor length, known as a transmission line model (Figure 4b). The crack opening displacement controls the effective resistance of the resistors. The electrode piezoresistivity attenuates the signal dissipation progressively along the capacitance length under strain and then creates a virtual length defined by the location where the signal is fully attenuated. We took advantage of this innovative mechanism of capacitance variation to create sensitive capacitive strain sensors that outperform conventional capacitive sensors based on the geometric effect (GF < 1). We simulated the response of our cracked sensor analytically and compared it with the geometric capacitance with the same sensor design without cracks (Figure 4c). The capacitance of the fragmented‐plate capacitor can be analytically studied using a model derived from the telegrapher's equations.^[^ ^34^ ^]^ Consequently, the transmission line capacitance is frequency and resistance/strain dependent and differs from the traditional (purely geometrical) capacitance equation by a term *g*(*f,R*) ranging between 0 and 1 according to *R* and *f* (more details on the model equations are given in the Supporting Information). Addressing the designed sensor geometries and parameters (frequency, initial capacitance, initial resistance, and resistance variation), we evaluated effective capacitive variation as a function of strain. Figure 4c shows that the transmission line capacitance decreases by >50% for only 1% strain (yellow zone), indicating GF > 50 for the low strain compared to GF < 1 for the response resulting from the geometrical effect. The desired consequence is a large shift in the resonance frequency for the global LC oscillator as seen in Figure 4d. A shift of 23 MHz/1% equivalent to 43% for the transmission line mechanism versus less than 0.5 MHz/1% (0.9% shifting) for the classical LC oscillator was observed, resulting in an enhancement of 50 times in the sensitivity. In addition, the LC wireless sensor with the fragmented‐electrode capacitator performed excellently as a strain sensor with high responsivity (≈1.3 MHz/<0.1%), providing readouts with high resolutions <0.1%, which could cover strain variations of composite structures.

### 2.4 Wireless Strain Detection and Sensor Integration in Composites

**Figure 5:**
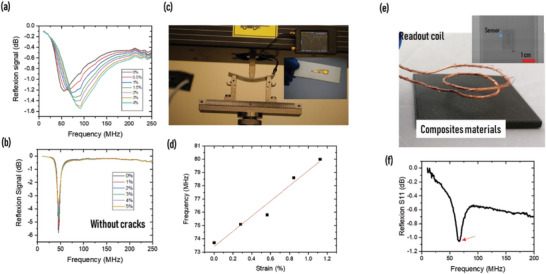
Ability of the cracked RFID sensor to detect low strain wirelessly: a) Signal reflection from the cracked RFID sensor, where a resonance frequency shift occurs under strain. b) Signal reflection from a noncracked RFID sensor, where almost no resonance frequency shift occurs under strain. c) Bending test of a GFRP specimen equipped with a cracked RFID sensor at its bottom (the inset shows the sensor at the bottom of the GFRP specimen). d) Response of the cracked RFID sensor to the strain resulting from the bending of the GFRP specimen. e) Image showing a piece of printed CFRP with an embedded cracked RFID sensor (the inset shows an X‐ray image of the implemented sensor). f) Signal reflection from the sensor implemented in the CFRP material, where the red arrow denotes the resonance frequency of the sensor.

The designed multilayer sensor was fabricated successfully via an innovative microfabrication process by using cleanroom technology, including sputtering and electroplating for metal depositions and photolithography and etching for structuration (the fabrication process is presented in detail in the Experimental Section and in Figure S6, Supporting Information). In addition, a sensor without cracks was tested for comparison (does not include a cracks initiator/Cr layer). The wireless response of the sensor was determined by measuring the reflexing signal (s11) between the reader coil and the sensor, allowing the acquisition of the resonance peak of the oscillating signal. The reader coil collecting signals from the sensor with a separation distance of 10 mm is shown in Video S1 (Supporting Information). The measured initial resonance frequency of ≈53 MHz matches very well with the calculated one for both sensors (with and without cracks), confirming the accuracy of the design and fabrication. Changing the capacitance of the LC circuit directly affects the sensors’ resonance frequency, as given by Equation S3 (Supporting Information). The result presented in **Figure** **5**a shows the ability to detect shifts in resonance frequency for very small strain (<0.5%) with a total shift of 38 MHz for 4% strain, equivalent to 71%. The resonant frequency of the cracked system increases with increasing tensile strain because of the transmission line mechanism contrary to the resonance frequency of a noncracked sensor, which decreases under strain owing to the geometric effect. This comparison clearly highlights the low sensitivity of the conventional sensor compared to our innovative sensor, where Figure 5b shows that almost no shifting appears during stretching of the noncracked sensor. However, the cracked sensor has good resolution and precision with the ability to detect strain levels lower than the fracture strain point of the composite materials that ranged between 1,5% and 2,5%. The observed results revealed higher values of sensitivity and resolution compared to reported RFID targets for strain sensing (Table S1, Supporting Information). In addition, the short response time (Video S1 and Figure S8, Supporting Information) of the cracked sensor paves the way for more applications, e.g., scanning of a large area containing a network of sensors by a While Unmanned Aerial Vehicles (UAVs) (Figure 1). The proposed sensor in this paper is specifically designed for near‐field sensing with a limited reading range. While UAVs may not directly support this type of sensing, the sensor can be combined with a minimized reader system mounted on a UAV to enable long‐distance measurements. Although near‐field sensors may face challenges on metal surfaces due to electromagnetic interference, however, utilizing specific designs and materials can help overcome this limitation. It's important to note that our research primarily focuses on non‐metallic applications, as metallic structures already have established inspection techniques, such as ultrasonic or electromagnetic methods. But based on the result, we can confirm that our sensor exhibits superior performance in terms of sensitivity, ability to detect very small strains, and response time compared to the other sensors. Specifically, our sensor demonstrated a high sensitivity of 50, which is significantly higher than the sensitivities reported for other sensors. In addition, our sensor was able to detect strains as small as 1%, which is much smaller than the detection limit of other sensors. Furthermore, our sensor exhibited a response time <10 ms, which is faster than the response times reported for most other sensors. Overall, these results demonstrate the potential of our proposed sensor for practical applications in various fields.

Because of their ability to wirelessly detect low strain, good response time, and precision, the proposed sensors are promising for tracking damages, such as delamination, cracks, or other defects, such as gas/liquid leakage in composite materials. Given the high sensitivity, soldering is not required as wireless power and data transmission becomes possible. The cracked RFID strain sensor is an excellent candidate for embedding inside composite structures without affecting the integrity of the materials. The wireless sensing system we developed utilizes electromagnetic waves for signal transmission. While electromagnetic interference (EMI) can pose challenges, we found that non‐interfering structures, such as glass fiber‐reinforced polymers, exhibited no difficulties in data collection. We chose to demonstrate the cracked sensor performance by monitoring the strain generated in GFRP during a bending test (Figure 5c). The cracked sensor was fixed on the bottom of a GFRP sample using epoxy adhesive and the strain response was measured wirelessly from the top side of the GFRP sample by real‐time continuous measurement of the resonance frequency of the sensor through a coil connected to a vector network analyzer (VNA). The low frequency targeted and the high conductivity of the antenna (coil) allow data collection with a very basic and cheap VNA. We continuously monitored the resonance frequency of the wireless system in real time using NanoVNA during the bending test. When the GFRP specimen was bent, the cracked strain sensor fixed on it was stretched, resulting in a decrease in capacitance of the sensing part of the sensor referring to the transmission line model. This caused an increase in the resonant frequency of the system, enabling the wireless monitoring of tensile strain on the sample during bending. For better understanding, the strain applied to the sensor can be deduced from the classical equation that relates the deflexion, *d*, with the strain, *ε*, for the three‐point bending test ($\varepsilon =6dh/L^{2}$), where *L* is the distance between supports and *h* is the thickness of the specimen. Figure 5d shows linearity between the calculated strain and the resonance frequency shift, confirming the high performance of the sensor in accurately monitoring very low strain.

Furthermore, we confirmed the possibility of integrating the sensor inside the composite materials while maintaining its ability to communicate with the external environment. Figure 5e shows a printed isotropic CFRP piece (0° orientation), including a cracked RFID sensor, as shown in the X‐ray image. Further, Figure 5f shows the reflected signal from the sensor inside the CFRP specimen, where the resonance peak appears clearly with a slight loss of the signal inside CFRP. Anisotropic CFRP is well known for electromagnetic interference (EMI) shielding, i.e., blocking and preventing radiofrequency (RF) waves from penetrating materials.^[^ ^35^ ^]^ To mitigate this effect, various strategies can be employed, including altering the sensor design to achieve a low resonance frequency, typically in the MHz range. This modification enhances signal propagation and minimizes the impact of EMI, enabling accurate strain detection within carbon‐fiber‐reinforced polymers. The integrity of CFRP after sensor integration was also tested and studied. We integrated our 50 µm sensor between the eight plies of CFRP oriented (0°90°). The sensor was covered by polyimide tape on both sides, which served as an insulating and protective layer to isolate active materials and avoid possible short circuit by the carbon fiber. The bending test revealed that the mechanical performance improved after sensor integration. This observation appears surprising initially; however, the reason is that the sensor acts as a sacrificial crack,^[^ ^36^, ^37^ ^]^ which helps to enhance the fracture toughness of the materials by introducing nonlocal extrinsic dissipation mechanisms. This is evident in Figure S5 (Supporting Information), where no degradation in strength and stiffness of the composite materials is observed after sensor implementation. Thus, this result confirms that the cracked RFID sensor is an excellent candidate for CSHM as it can be embedded in composite structures without affecting the integrity of the material as well having the ability to accurately follow the strain in the structure even on a large scale by enabling a network of wireless sensors (pipelines of several kilometers, large gas tanks, aircraft, etc.), Video S2 (Supporting Information) shows the proof of concept of some cracked sensors embedded in CFRP laminated plates.

## 3 Conclusion

We demonstrate the potential of RFID technology to realize ultrasensitive wireless strain sensors that can detect strain from inside composite structures. We realize an ultrasensitive integrable wireless sensor with wireless communication, low consumption, easy installation, low cost, and consistent reliability compatible with the requirements of composite structures. Original methods have been developed to increase the sensitivity of the RFID strain sensor based on creating a pattern of nanocracks in the capacitive part of the passive LC circuit, which forms a transmission line model in the capacitor. The transmission line model creates a virtual length of the capacitor according to the piezoresistivity of the electrodes. This virtual length transforms into a large capacitance variation even for small strains, leading to a shift in the resonance frequency of the LC oscillator. We maximized the coupling between the sensor and the reader, as well as selected the appropriate sensor resonance frequency by optimizing the sensor design. For the first time, an RFID strain sensor capable of wirelessly detecting lower strain than the fracture point strain of composite materials (≈1.5%) has been fabricated and fully tested. We confirm the ability of our sensor to send strain data through GFRP and CFRP when embedded inside materials and conduct a bending test that shows an excellent reflection of the real strain inside the materials. Non‐intrusive and non‐degradation of the mechanical properties of the materials are features of the new RFID strain sensor, where we validate that no significant degradation of stiffness and strength appears in the materials when sensors are embedded. This technology is ready for transfer to WSN systems through the addition of a network of sensors in a large structure; an example of several kilometers of pipelines is proposed to solve a big problem of pipeline monitoring and reduce the enormous cost of oil and gas structural monitoring that depends on classical NDT methods. One drone‐containing readout system connected to a Wi‐Fi system can read the WSN data and send it directly to a data treatment station, which can help avoid catastrophic failure and defect in composite structures and be the next generation of SHM systems.

## 4 Experimental Section

### Fabrication Process of the Cracked LC Sensors

Microfabrication technology was used to pattern the metallic LC circuit on a flexible substrate. The fabrication of RFID sensors was realized on 4 inch wafers, with each wafer having the capacity to produce a set of seven sensors (Figure S6a, Supporting Information). The first challenge was to adapt the flexible substrate to microfabrication tools, which were usually designed to suit rigid substrates, such as silicon or glass wafers. Any flexible substrate material (PET, PEN, PI, etc.) and thickness were suitable for the fabrication process; however, for the sensor, a 50 µm polyimide (PI) film was used with excellent thermal and mechanical properties and adapted to such applications (low strain detection). First, a piece of polyimide (DuPont Kapton HN) was cut in the dimensions of the silicon wafer. After cleaning the 4 inch polyimide substrate by rinsing in acetone and isopropanol, the polyimide substrate was fixed on the silicon wafer by adding a specific oil in between. To avoid air bubbles and delamination during the curing step, the polyimide substrate fixed on the wafer was placed under vacuum for 10 min. The LC circuit was patterned on the two sides of the polyimide substrate (Figure S6b, Supporting Information), with the top electrodes and the coil on one side (top) and the bottom electrode with the coil capacitor connection on the other side (bottom). The patterns were realized layer by layer using both metal etching and additive processes in four successive steps. The first step involved patterning the circular planar coil on the top side by using a wet‐etching process. The coil should be thick to avoid losses in the electromagnetic reflection–transmission signal. Accordingly, electroplating tools (CEMCON 1500) were used to deposit a thick gold layer (2 µm) over the entire substrate surface. Other chipper materials, such as Al or Cu could be used; however, the adhesion between these metals and the substrate should be improved beforehand by adding some interlayer or changing the roughness of the PI surface. Realizing a reactive ion etching process by attacking the surface with oxygen plasma could be a solution to improve the adhesion between metals and PI.^[^ ^38^ ^]^ The photolithography process was used to fabricate the circular planar coil. A positive photoresist (AZ 5214 E photoresist) was deposited by spin coating on the top of the metal layers with a thickness of 1.6 µm and then cured at 110° for 2 min. By using an ultraviolet (UV) contact aligner (EVG6200), 80 J s^−1^ was injected into the photoresist through a mask, fabricated locally with a laser writer (DWL2000), to structure the photoresist. The resist was developed by using the AZ 726 MIF Developer for 1 min to form the final designed structure of the top electrodes. At this stage, the metal‐etching process was executed by rinsing the entire wafer in a gold‐etching solvent from ALDRICH for 30 min. Then, the wafer was rinsed in a resist remover (NMP) to remove all the resist and obtain a total of seven Au coils on the same substrate, as shown in Figure S6 (Supporting Information). Note that the L–C connection on the bottom side was fabricated the same way as the alignment to superpose the two layers. Nevertheless, the top electrodes were patterned on the same side as the coil before turning the PI substrate and creating the L–C connection. The capacitor electrodes were designed in rectangular shape with a length of 10 mm and width of 3 mm opposite to each other and separated by a PI layer as the dielectric. The etched electrodes were made of a 60 nm chrome layer and 100 nm gold layer superposed with each other. A lift‐off process was performed to pattern the electrodes to avoid coil etching as both were fabricated using the same materials. AZ 5214 was used as a negative resist to facilitate the lift‐off process and avoid the adhesion between the resist and metals in the removal phase. The negative resist was created by curing the spin‐coated AZ 5214 film at 105° for 2 min, and after exposing the target zone to UV radiation, the resist was inverted by post‐curing at 120° for 2 min and then exposing all the surfaces to UV radiation at 200 J s^−1^ for 1 min. Note that the coil layer should be aligned with the superposed electrode layer in this step. AZ 726 MIF Developer was used again for emptying the electrode zone. This step was followed by sputtering the Cr and Au layers and then removing all the surrounding resist by using NMP remover to form electrodes connected to the coils. The same process was repeated on the other side to create the bottom electrodes. At this stage, the PI substrate was removed from the silicon wafer and cleaned with acetone, isopropanol, and water. The final sensor design was cut with a laser‐cutting machine (Universal Laser Systems). Finally, the top and the bottom sides were connected by piercing a small hole with a laser and filling it with conductive epoxy (CW2400 silver conductive epoxy from CircuitWorks) to obtain a connected LC circuit on a flexible substrate.

### Creation of the Network of Nanocracks and Resistance Measurement of the Electrodes

Introducing the cracks in the metallic layers was necessary to achieve sensitive sensors. These cracks were created by load–unload cycling. Each sensor was subjected to 3000 cycles of 4% strain with a rate of 0.1 mm s^−1^ by using 5944 Instron universal testing frame. To follow the progress of the crack multiplication during the cycling loading, the resistance of the electrodes was measured (which was strongly correlated to the number of cracks) with KEYSIGHT 34461A digital multimeter, and the connection was realized by fixing the wire at the edge of the electrodes by using conductive epoxy (CW2400 silver conductive epoxy from CircuitWorks).

### Electrical Characterization of the Cracked Sensor

After cycling, a static stretching test was performed on each sensor to ascertain the resistance behavior of the electrodes. The strain was applied by a small tensile machine (Kammrath & Weiss), and the resistance was measured in real time through a microprobe (KRN‐09S), where the probe was fixed at the edge of the electrodes. The electrical characteristics (coil inductance, capacitance, and coil resistance) were measured with a high‐frequency LCR meter (Agilent E4980A) that could sweep up to 3 GHz. The measurement was performed in static mode by sweeping in a frequency around the system's resonance frequency. The measurement was realized through a physical wire to avoid noise in the probe.

### Wireless Signal Detection of the Sensor

The sensor data were read remotely by RFID technology, i.e., by electromagnetic coupling between the transmitter and the receiver. In this case, no direct power source was required to power the sensor, and it was powered wirelessly from the transmitter. However, the transmitter could be a simple high‐frequency oscillator that sweeps across a frequency band generating radio frequency (RF) power. A VNA (Agilent N5225A) was selected for the test, which could measure the reflection parameter (S11), represented by the reflected signal power over the incident power.

The communication between the sensor and the VNA was realized through an inhouse‐fabricated antenna (readout coil), which was fabricated by using two circular turns of copper wire of 20 mm diameter connected to an SMA connector and covered by 25 µm polyimide tape from both sides to ensure the insulation of the coil. When the sensor was brought in close proximity to the VNA, it absorbed a portion of the RF power only at its resonant frequency, and then a reflection signal (S11) of the oscillator circuit was active only at the resonance frequency, where a peak related it with negative S11. Further, an economical and handheld VNA (NanoVNA) was used to replace the lab VNA equipment and afforded the same result, where it was succeeded in obtaining the shift in resonance frequency by measuring S11 between the sensor and readout coil.

### Wireless Strain Detection and Bending Test

The strain was applied directly to the sensor by a small tensile machine (Kammrath & Weiss), where a very small elongation could be applied (<20 µm). A 100 µm stretching step was applied to the sensor for each collecting resonance frequency shift. The elongation ∆*L* was transformed into the strain *ε* by dividing it by the total length of the stretched zone *L*_0_. Further, the readout coil was fixed 10 mm from the sensor and harvested the sensor response under strain.

### Bending Test

To observe the composite material strain wirelessly, the sensor was fixed at the bottom of a GFRP specimen (manufacturing process presented in the section below) and then a bending flexural test was performed. The GFRP specimen was mounted between two supporting pins, and a force was applied by a loading pin using a universal testing machine (Instron 2885). The specimen was bent under uniaxial bending stress to create a strain in the capacitive part of the sensor. The response of the sensor under strain was read through the GFRP materials by the NanoVNA system.

### Printed CFRP Specimen Including Sensors

The sensor was embedded in a small CFRP panel printed with a 3D printer and composite materials from 9T LABS. The X‐ray image of the sensor inside the CFRP panel was acquired with Xtek XT H225.

### CFRP Specimen Preparation

The CFRP specimen was prepared by using unidirectional tape prepreg carbon/epoxy (T700/M21 Hexply, Hexcel). Eight plies of 0.25 mm thickness were stacked by using the hand lay‐up technique to produce adherents with 2 mm thickness and [0°]_8_ stacking sequence. The sensors were distributed in the panels between the second and third plies. The 300 × 300 mm panels were cured by compression molding. A hydraulic hot press was used to compress the sample at a pressure of 7 bar and 180°C for 2 h. Finally, the sample was cut from the fabricated panel by using an abrasive water jet machine. The panel was cut into 10 pieces of 120 × 50 mm, with half of them, including sensors.

### GFRP Specimen Preparation

The thermoplastic composite material used for the bending test was impact‐modified polypropylene copolymer (IPP) reinforced with continuous E‐glass fibers. The composite was provided in the form of a roll of 110 mm‐wide unidirectional tape with a thickness of 0.25 mm. The provided composite included a nominal volume fracture of 45% of E‐glass fibers. A customized mold technique was used to produce 120 × 50 mm laminates by stacking tapes in a sequence and consolidating them under a temperature cycle with a cooling cycle of (22°/min) and pressure of 7.5 bar. Then, the sensor was installed at the bottom of the GFRP specimen epoxy following the direction of the strain resulting from bending the specimen.

## Author Contributions

H.N. and G.L. conceptualized the study. H.H. contributed in the experiments. All authors contributed to writing and editing the manuscript.

## Conflict of Interest

The authors declare no conflict of interest.

## Supporting information

Supporting Information

Supplemental Video 1

Supplemental Video 2

## Data Availability

The data that support the findings of this study are available in the supplementary material of this article.
