# Supplementary material for: High‐Sensitivity RFID Sensor for Structural Health Monitoring
Source: Adv Sci (Weinh). 2023 Jul 5;10(26):2301807. doi: 10.1002/advs.202301807 (PMC10502838; doi:10.1002/advs.202301807)
Supplement: Supplementary file 1 — Supporting Information [file ADVS-10-2301807-s003.pdf]

ADVANCED SCIENCE
Open Access

Supporting Information

for *Adv. Sci.*, DOI 10.1002/advs.202301807

High-Sensitivity RFID Sensor for Structural Health Monitoring

Hussein Nesser, Hassan A. Mahmoud and Gilles Lubineau**

Supporting Information for “High-sensitivity RFID sensor for structural health monitoring”

Hussein Nesser, Hassan A. Mahmoud, and Gilles Lubineau**

Mechanical Engineering Program, Physical Sciences and Engineering Division, King Abdullah University of Science and Technology (KAUST), Physical Science and Engineering Division, Thuwal 23955-6900, Kingdom of Saudi Arabia

Mechanics of Composites for Energy and Mobility Laboratory, King Abdullah University of Science and Technology

E-mail: h_nesser@hotmail.com, gilles.lubineau@kaust.edu.sa

Keywords: Structural health monitoring, Radio frequency identification device, Chip less sensors, wireless strain monitoring, Strain sensor

1. Performances of available RFID strain sensors

Table S1: Performance and limitation of the conventional chip less wireless strain sensors in terms of sensitivity and resolution

**Publication**	**Application**	**Strain range**	**Resolution**	**GF**
[1]	Skin (human motion)	0%–50% strain	5%	0.6
[2]	Air tubes	0%–24%	2%	0.5
[3]	Medical application	0%–50%	0.2%	2
[4]	Health monitoring	0%–15%	5%	0.11
[5]	Skin (human motion)	0%–40%	10%	0.67
Our Work	SHM	0%-5%	0.1%	50

2. Design of RFID sensors with cracked electrodes

The LC sensor includes a capacitance C and an inductance L, where C and L both depend on the geometry. The capacitance C is calculated as follows [6]:

$$C=\varepsilon_0\varepsilon_r\frac{l\omega}{d}, \qquad (S1)$$

where ε_0 and ε_r are the vacuum permittivity and permittivity of the dielectric material, respectively, l is the electrode length, ω is the electrode width, and d is the distance between the two electrodes (dielectric material thickness).

The inductance L for a planar coil is defined as follows [7]:

$$L=K_1\mu_0\frac{n^2 d_{avg}}{1+K_2\delta}, \qquad (S2)$$

where $d_{avg}=\frac{d_{out}+d_{in}}{2}$ and $\delta=\frac{d_{out}-d_{in}}{d_{out}+d_{in}}$; d_{out} and d_{in} are the outer and inner coil diameters, respectively, as presented in figure S1. μ_0 is the magnetic permeability of free space, n is the number of turns. K_1 and K_2 are both layout-dependent and have values of 2.34 and 2.75, respectively, for planar circular inductors.

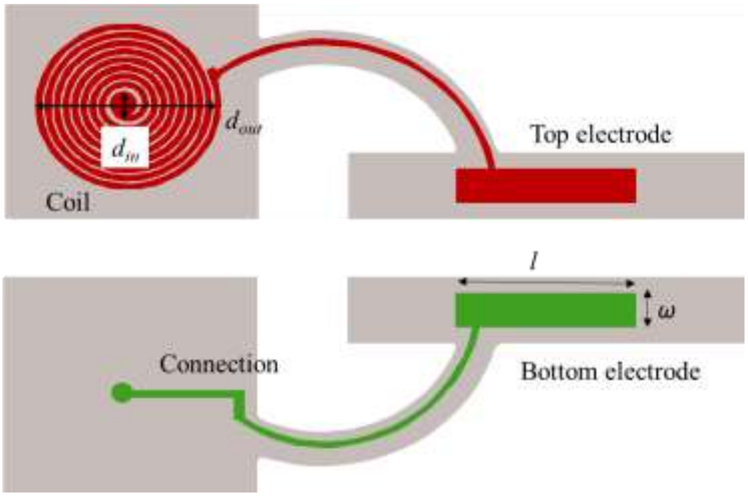

Figure S1: Sensor design where the top face of the substrate (gray) includes the coil and one electrode and the bottom face of the substrate includes the connection and the other electrode.

The sensor performances, including resonance frequency f_r electromagnetic response α, mutual coupling M, and loss factor η are dependent on the geometry of the sensor and their electrical parameters (C and L). Some analytical models can predict the relation between sensor characteristics and geometry, enabling the simulation of sensor design before fabrication.

The resonance frequency of the LC oscillator is the most important factor as the strain data collected from the sensor are related to resonance frequency shift. In addition, the initial resonance frequency determines the crack density required in the electrodes. The resonance frequency of the LC oscillator is well known and defined as follows [6]:

$$f_r = \frac{1}{2\pi\sqrt{LC}}. \tag{S3}$$

Furthermore, the loss factor η is defined as the ratio between the coil resistance (R_{coil}) and the frequency-dependent (f) inductance (L) as follows:

$$\eta = \frac{R_{coil}}{2\pi f L}. \tag{S4}$$

Minimizing this factor was one of our objectives to maximize the signal transmitted back to the reader. If the coil resistance is high, the sensor coil loses all the signal emitted from the reader and the signal is totally dissipated in the sensor. In such a case, there is no possibility of reading the shifting of the resonance frequency. Accordingly, reducing the coil resistance was one of our challenges during the sensor design, as the only possibility of highly reducing the coil resistance while maintaining high inductance is by creating a large cross section of the coil wire, as given by equation S5:

$$R_{coil} = \rho\frac{l}{A}, \tag{S5}$$

where ρ is the material conductivity, l is the length of the wire in the coil, and A is the cross section, which equals $w \times t$, with w and t being the width and the thickness of the coil wire, respectively.
We increased the coil thickness to 1.5 µm to achieve a resistance of 7.5 Ω, small enough to minimize the loss factor of the coil and maintain the electromagnetic wave.

The mutual inductance M quantifies the magnetic field or flux that links both coils and relates directly to the distance between the two coils and their properties (radius and inductance):

$$M = k\sqrt{L_1 L_2}, \tag{S6}$$

where

$$k(z)=\frac{r_1^2 r_2^2}{\sqrt{r_1 r_2}\left(\sqrt{z^2+r_2^2}\right)^2}. \qquad (S7)$$

k is the coupling coefficient between the coils and L_1 and L_2 are the inductances of the reader and sensor coil (H), respectively. As shown in figure S2, r_1 is the radius of the reader coil, r_2 is the equivalent radius of the sensor inductor, and z is the coupling distance between the two coils.

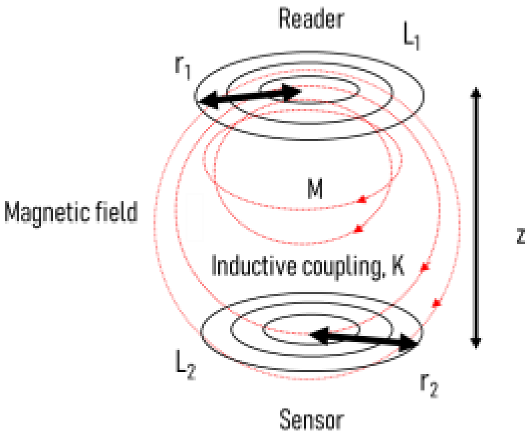

Figure S2: Electromagnetic coupling between two planar coils, where the mutual coupling M is dependent on the distance z between the two coils; the red line represents the magnetic field generated by the reader.

Optimizing the coupling distance is another challenge in achieving an effective sensor capable of communicating from inside a composite structure. The range of the passive RFID reading distance ranges from a few millimeters to a few centimeters. Our target was to achieve strain detection wirelessly from a distance of 2–3 cm. This could be achieved by optimizing the two antenna parameters and developing an adapted interrogation system suitable for our sensor.

Table S2 summarizes the sensor capacitor and coil dimensions deduced from the analytical model based on the previous equations. Further, the selected sensor dimensions resulting from the sensor parameters are listed in **table S3**. The sensor fabricated according to the selected dimensions showed similar results for electrical parameters.

Table S2: Characteristics of the coil and capacitor in the fabricated sensor

**Sensor coil**		**Sensor capacitor**	
Conductive height, *h*	1.5 µm	Length	10 mm
Conductive width, *t*	300 µm	Width	3 mm
Spacing, *s*	200 µm	Cr thickness	60 nm
Diameter, d_{out}	10 mm	Au thickness	100 nm
No. of turns, *n*	8	Dielectric thickness	50 µm

Table S3: Sensor characteristics based on the analytical study

C_0	R_0 (electrodes)	R_{coil}	L_0	R (0%–4%)	Resonance frequency	Quality factor
45 PF	100 Ω	7.5 Ω	0.21 µH	100 Ω–10 KΩ	50 MHz	0.113

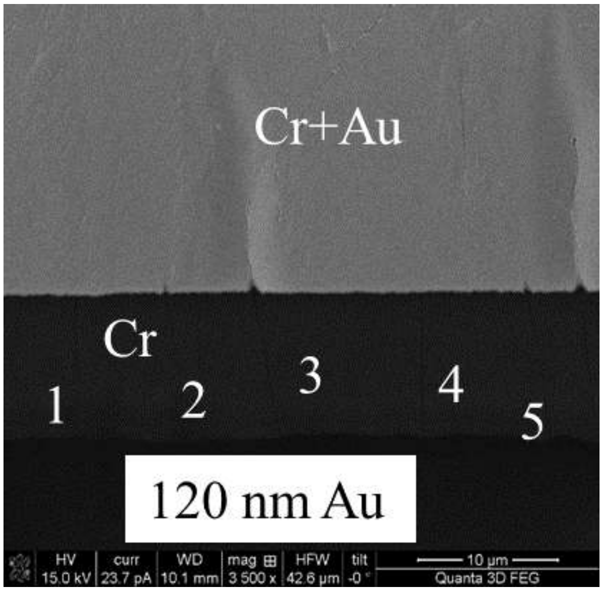

Figure S3: SEM image showing the inability of cracks in the Cr layer (dark color) to propagate in the thick gold layer even after high cycling strain.

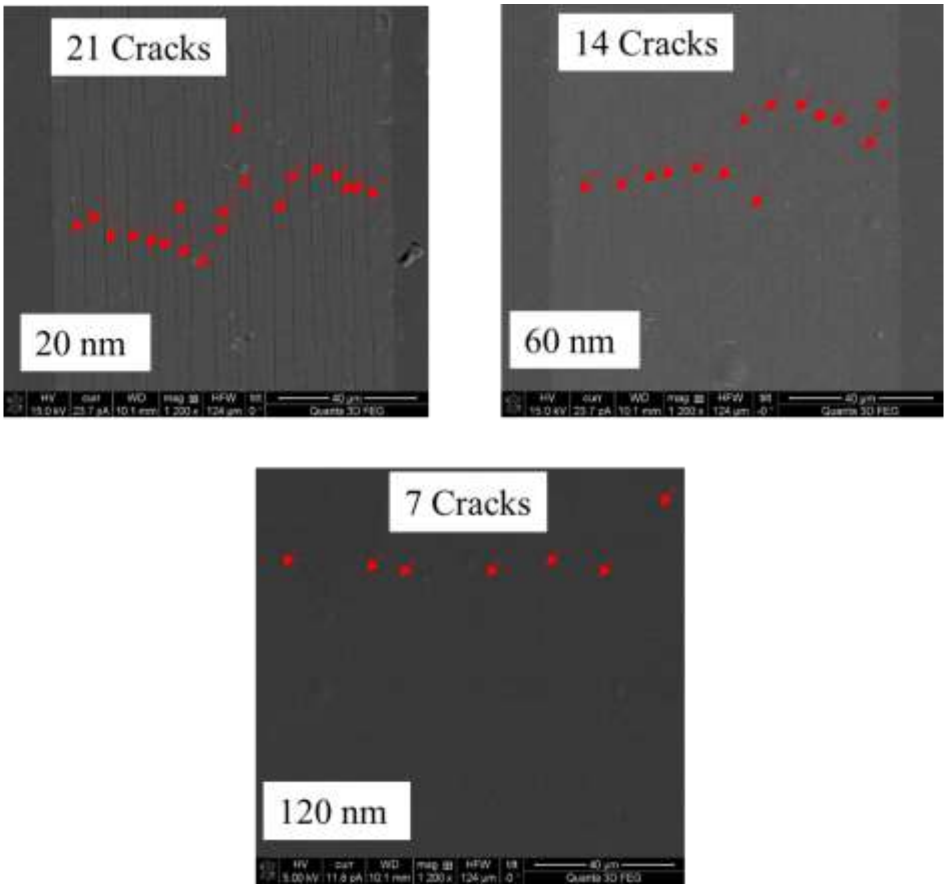

Figure S4: SEM image showing the number of cracks in a zone of 100-µm width of Cr/Au with 60-nm Cr thickness and 20-, 60-, and 120-nm Au thickness.

3. Capacitance equation for transmission line mode

According to the transmission line model, the voltage dissipation in the sensor creates a "virtual" sensor length (represented by the distance from the origin to the point where the signal is fully attenuated). Analytically, the virtual capacitance is derived from the telegraph equation that defines the voltage attenuation along the transmission line (sensor length) as follows [8]:

$$V(z) = V_0 e^{-\sqrt{\pi f R C} z}, \tag{S8}$$

where V_0 is the magnitude of the alternative input voltage and the exponent is the attenuation factor $\alpha = \sqrt{\pi f R' C'}$. In this equation, f is the frequency of the interrogation signal, C′ is the capacitance per unit length, and R′ is the resistance per unit length. The effective/virtual capacitance (C_{eff}) derived from **Equation 8** can be expressed as follows [9]:

$$C_{eff} = C_0(1+\varepsilon)g(f, R(\varepsilon)). \tag{S9}$$

The sensor's effective capacitance is affected by an additional strain-frequency dependent term $g(f,R)$ representing the transmission line effect. ε is the external strain, the term $(1 + \varepsilon)$ represents the geometrical variation under stretching, and $R(\varepsilon)$ is the strain-related electrode resistance.

g(f,R) is a variable factor ranging from 0 to 1 depending on frequency f and strain ε. It was concluded in our previous work [9] as

$$g(f,R) = \frac{-\ln\left(\frac{V_{min}}{V_0}\right)}{\sqrt{\pi f C R}}, \tag{S10}$$

where V_{min} is the voltage at which the transmission line becomes ineffective (the signal does not reach the end of the sensor anymore/ "nonexistent" voltage).

Equation 9 highlights a new relationship between the strain and effective capacitance. This is not only a purely geometrical effect as in the classical capacitor strain sensor (for which $C = C_0(1+\varepsilon)$) but also has an additional frequency and resistive term $g(f,R)$.

4. Integrity of the composite materials after RFID sensor implementation

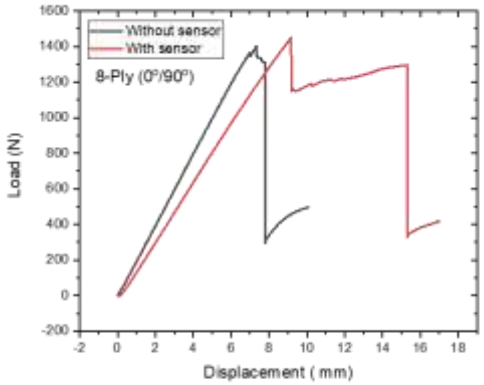

Figure S5: Load–displacement curve of two CFRP specimens of 8 ply, one with an embedded sensor (red curve) and another without a sensor (black curve). The result shows that there is no noticeable degradation in strength and stiffness after sensor implementation.

5. Fabrication process of RFID sensors

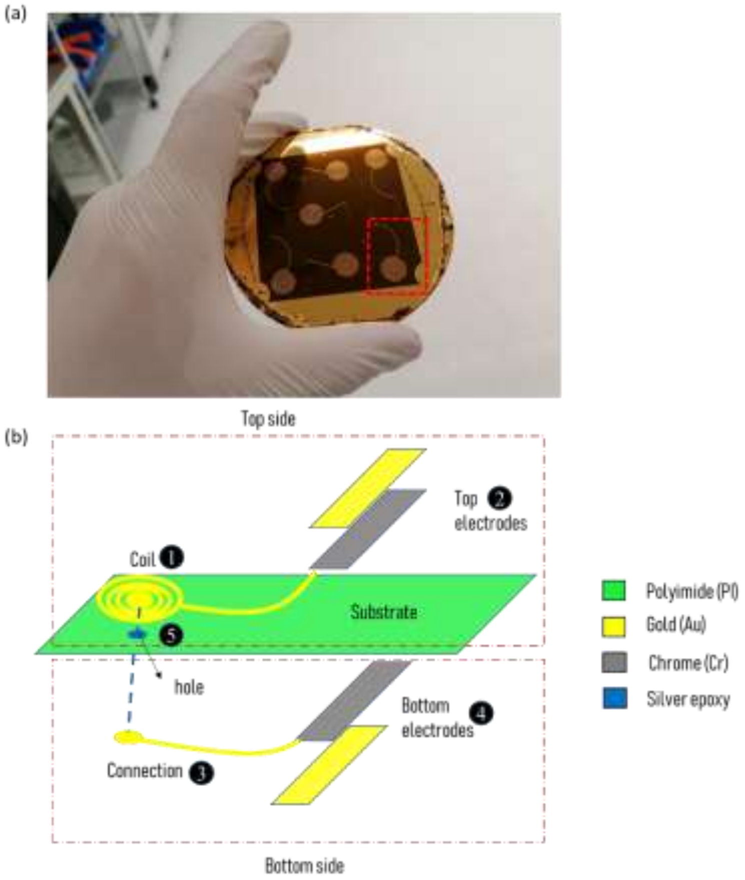

Figure S6: (a) Silicon wafer covered by polyimide film during the RFID sensor fabrication process and (b) RFID sensor layers showing multifabrication steps at the top and bottom of the substrate.

6. Additional data

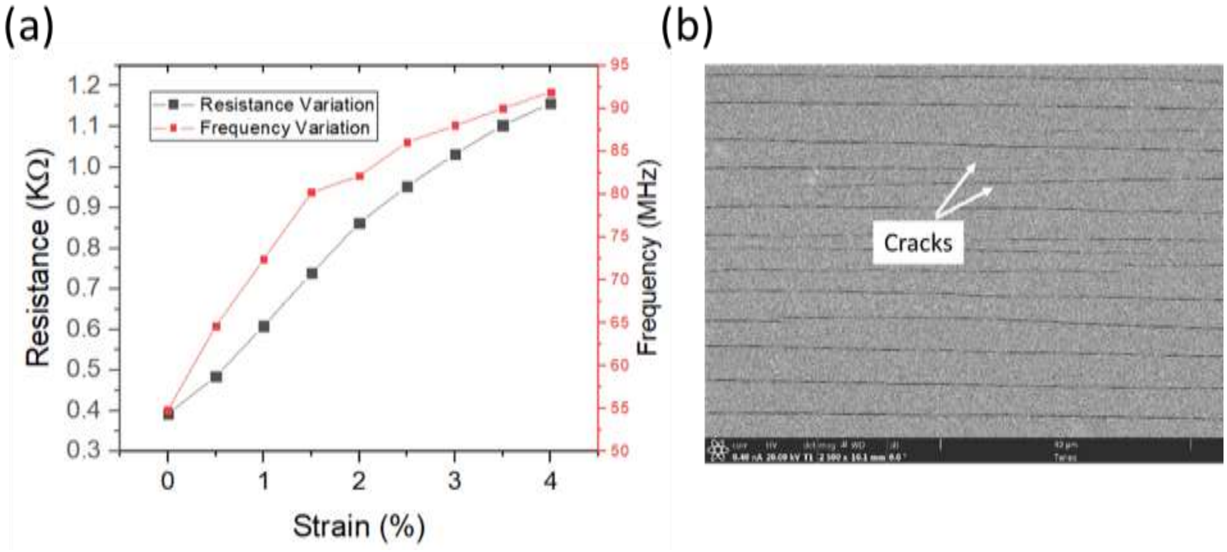

Figure S7: (a) Resistance versus strain of the electrodes of a wireless strain sensor with the following configuration: Cr thickness of 60 nm and Au thickness of 100 nm, frequency versus strain for the same cracked LC sensor. (b) SEM image for the cracks generated in the Au layer.

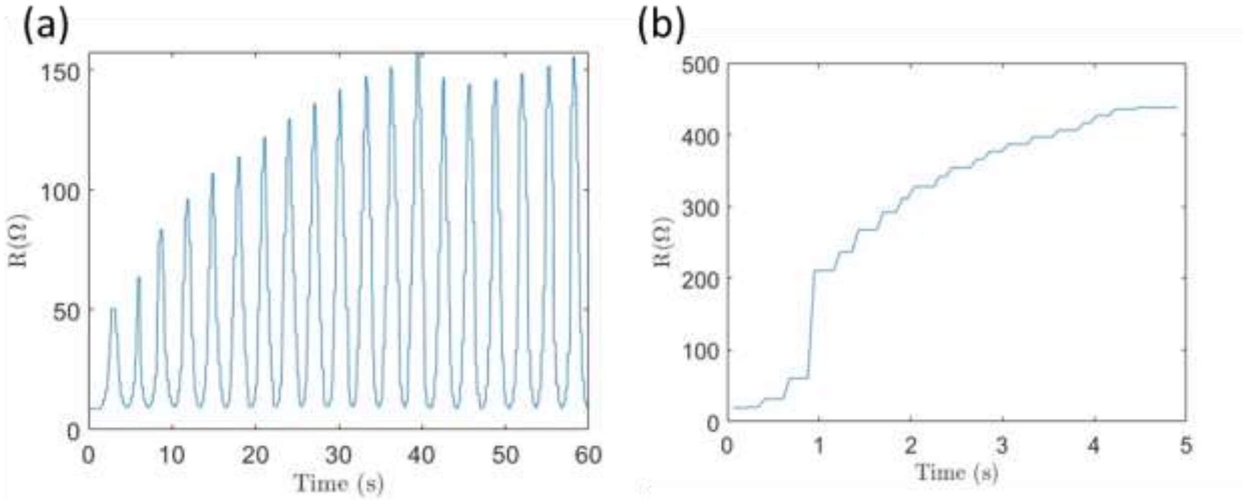

Figure S8: Sensor Response Times under (a) Cyclic Test (b) Static Test

1. Niu, S. *et al.* A wireless body area sensor network based on stretchable passive tags. *Nat. Electron.* **2**, 361–368 (2019).
2. Min, S.-H. *et al.* Stretchable chipless RFID multi-strain sensors using direct printing of aerosolised nanocomposite. *Sens. Actuators Phys.* **313**, 112224 (2020).
3. Lee, J. *et al.* Stretchable and suturable fibre sensors for wireless monitoring of connective tissue strain. *Nat. Electron.* **4**, 291–301 (2021).
4. Huang, X. *et al.* Materials and designs for wireless epidermal sensors of hydration and strain. *Adv. Funct. Mater.* **24**, 3846–3854 (2014).
5. Dong, W., Cheng, X., Wang, X. & Zhang, H. Fractal serpentine-shaped design for stretchable wireless strain sensors. *Appl. Phys. A* **124**, 1–6 (2018).
6. Nesser, H. & Lubineau, G. Strain sensing by electrical capacitive variation: From stretchable materials to electronic interfaces. *Adv. Electron. Mater.* **7**, 2100190 (2021).
7. Mohan, S. S., del Mar Hershenson, M., Boyd, S. P. & Lee, T. H. Simple accurate expressions for planar spiral inductances. *IEEE J. Solid-State Circuits* **34**, 1419–1424 (1999).
8. Rizzello, G., Naso, D., York, A. & Seelecke, S. A self-sensing approach for dielectric elastomer actuators based on online estimation algorithms. *IEEEASME Trans. Mechatron.* **22**, 728–738 (2016).
9. Nesser, H. & Lubineau, G. Achieving Super Sensitivity in Capacitive Strain Sensing by Electrode Fragmentation. *ACS Appl. Mater. Interfaces* **13**, 36062–36070 (2021).
